# Larval environment influences vector competence of the malaria mosquito *Anopheles gambiae*

**DOI:** 10.5281/zenodo.10798340

**Published:** 2016-06-29

**Authors:** Antoine M.G. Barreaux, Priscille Barreaux, Kevin Thievent, Jacob C. Koella

**Affiliations:** a Laboratory of Ecology and Epidemiology of Parasites, Institute of Biology, University of Neuchâtel, Rue Emile-Argand 11, 2000 Neuchâtel, Switzerland; b Merkle Lab, Department of Entomology, Pennsylvania State University, University Park, PA, 16802, USA

## Abstract

**Background:**

While environmental factors such as temperature can influence the vector competence of mosquitoes directly, for example by affecting the longevity of the mosquito and the development of the malaria parasite they may also have an indirect impact on the parasite’s transmission. By influencing larval development, they may affect the adult traits that are important for the parasite’s development and transmission. We studied the influence of two larval environmental factors, food availability and temperature, on the probability that mosquitoes infected with the malaria parasite survived to harbour sporozoites in their salivary glands.

**Materials and methods:**

*Anopheles gambiae* larvae were reared at 21ºC, 25ºC or 29ºC, and fed either a standard larval diet or half of it. Adults could blood feed on mice harbouring the infectious gametocytic stage of *Plasmodium berghei* ANKA transformed with green fluorescent protein (GFP). Survival was assessed every 24 hrs up to 21 days post infection, when surviving mosquitoes were dissected to check the salivary glands for sporozoites with a fluorescent microscope sensitive to GFP. Using a binomial GLM we analysed ‘vector competence’, i.e. if mosquitoes survived until dissection and harboured sporozoites in their salivary glands.

**Results:**

Vector competence dropped by about a third if we fed larvae half the standard food regime. The effect of temperature during the larval period depended strongly on the food regime. At low food, increasing temperature from 21ºC to 29ºC increased vector competence from about 0.18 to 0.48, whereas at standard food, vector competence dropped from about 0.67 at 21ºC to 0.56 at 29ºC.

**Conclusions:**

Thus, perceptions and models about the role of environmental change on the transmission of malaria should include how the environment changes adult life-history by influencing larval development.

## 1 Introduction

The competence of a vector to transmit an infectious disease is the result of a complex interplay between parasite and vector traits, and how the environment influences these. Rising temperature, for example, is expected to enable the parasite to develop more rapidly inside the mosquito [1–3], but may decrease its chance of surviving its developmental period [1,4], and it can shorten vector longevity [5]. Depending on the details of the interactions between these traits, increasing temperature can, overall, increase or decrease vector competence [6–11]. Nutrition also greatly influences vector competence [12] by affecting the infection load [13], the immune response [14] and the longevity [15,16] of the vector.

In addition to such direct effects, the environment may influence vector competence indirectly by affecting larval development [17–19], thus having a carry-on effect on the adult traits underlying vector competence [20,21]. Food and temperature during larval development, for example, influence the longevity of adult mosquitoes [22], and larval temperature can influence the transmission of malaria [23] and Chikungunya [24]. Part of this indirect effect may be a simple consequence of size: larval food [21] and temperature [25] influence adult size, which in turn influences the probability of infection [25], the longevity of mosquitoes [22,26] and the survival of infected mosquitoes [25]. To better understand the complex interactions between the larval environment, larval development, adult size and vector competence, we studied the combined effect of temperature and food during larval development on the probability that the mosquito *A nopheles gambiae* survives infection by the malaria parasite *Plasmodium berghei* and harbours sporozoites in its salivary glands.

## 2 Materials and methods

We used the Kisumu strain of *A n. gambiae s.s.* [27]. Newly hatched larvae were placed individually in 12-well-plates containing 3 ml of deionised water, to which we added Tetramin**TM** baby fish food daily. The mosquitoes were reared at 21ºC, 25ºC or 29ºC, and fed either a standard larval diet or half of the standard. The standard diet at 25ºC and 29ºC was 0.04 mg per larva on the day of hatching, 0.06 mg for 1-day-old larvae, 0.08 mg for 2 day olds, 0.16 mg for 3 day olds, 0.32 mg for 4 day olds, and 0.6 mg for 5 day old and older larvae. At 21ºC pupation is about 3 days later in our lab than at the higher temperatures (unpublished data). We reduced daily standard food at this temperature to achieve about the same total amount of food during larval development (day of hatching: 0.04 mg per larva, 1 day old: 0.05 mg, 2 days old: 0.06 mg, 3 days old: 0.08 mg, 4 days old: 0.12 mg, 5 days old: 0.19 mg, 6 days old: 0.32 mg, 7 days old: 0.38 mg, 8 days old: 0.45 mg; 9 days old and older: 0.45 mg). Each pupa was put into a 180 ml plastic cup covered with mosquito netting. After emergence, males were discarded and females were given access to 10% sugar solution at 25±1ºC. As such, the adult environment was identical for all cohorts of mosquitoes.

### 2.1 Infection

Sugar was removed from the cups 24 hrs before the infectious blood meal, when adult mosquitoes were about 4 days old. For each food and temperature treatment the mosquitoes were grouped into four cups (with close to equal numbers per cup; see Table 1), which were randomly allocated to four mice harbouring the infectious gametocytic stage of *Plasmodium berghei* ANKA transformed with green fluorescent protein (GFP, obtained from the Institute of Cell Biology, University of Bern, Switzerland). We allocated to each mouse one fourth of the mosquitoes of each combination of food and temperature (one cup) to spread the effect of the potential differences among mice across all treatments. The mice were anaesthetised by intra -peritoneal injection of 8.5 ml/kg of a mix of Xylazine Xylasol® (solution: 20 mg/ml), Ketamine Ketasol® (solution: 100 mg/ml) and PBS (phosphate buffered saline) and were placed on the cups containing the mosquitoes for 10 min. One day after the blood meal, female mosquitoes that were fully fed (292 out of 402) were put individually in cups with 10% sugar solution and kept at 19±1ºC, since higher temperatures block the development of the parasite. Unfed mosquitoes were removed from the experiment.

**Table 1.** Sample size for mosquito infections.

	Food	Blood fed	Blood Non fed	Total
21 °C	Standard	49	21	70
	Low	16	48	64
25 °C	Standard	67	6	73
	Low	50	11	61
29°C	Standard	58	21	69
	Low	52	3	55

### 2.2 Dissection

Survival was assessed every 24 hrs up to 21 days after infection, when all surviving mosquitoes (185 mosquitoes) were dissected in LOCKE solution [28]. The wings were measured from the tip to the distal end of the alula (excluding the fringe) [29] with the software Image J (version 1.47f7); we used the mean length of the two wings for analyses. We dissected the salivary glands out of the mosquito and isolated these in LOCKE solution on a microscope glass slide (magnification x32). These were then checked for the presence of sporozoites with a fluorescent microscope sensitive to GFP.

### 2.3 Data analysis

Wing length was analysed with an ANOVA including larval temperature, larval food and their interaction. Data were tested for normality with a Shapiro test and for homoscedasticity of the variance with Bartlett tests. All other analyses were binomial GLMs. Each one included larval temperature, larval food and their interaction and wing length as a covariate. We analysed three outcomes: survival up to the time of dissection, infection success (i.e. whether we found sporozoites in the salivary glands, considering only the mosquitoes that had survived up to dissection), and ‘vector competence’ (mosquitoes were classified as competent if they survived until dissection and harboured sporozoites in their salivary glands). We defined vector competence this way based on the definition in the glossary of the United States Department of Agriculture, National Agricultural Library 2015: ‘vector competence’: The physiological ability of a vector organism to acquire, maintain and transmit an infectious agent, as described by susceptibility to a pathogen, immune response, and sustaining infection long enough for disease transmission to occur. Vector competence is therefore both the probability to survive long enough for transmission to occur (long enough to acquire sporozoites in the salivary glands) and the probability of having the sporozoites in the salivary glands. All analyses were performed with R 3.0.2.

## 3 Results

### 3.1 Mosquito size

Wing length decreased from 3.33 mm (±0.002 SE) in mosquitoes that had been reared at 21ºC to 3.21mm (± 0.002 SE) at 25ºC and 2.99 mm (± 0.002 SE) at 29ºC, (F=99.6; P<0.001). Wings were longer, if larvae had obtained the standard diet (3.26 mm ± 0.001 SE) than low food (2.99 mm ± 0.001 SE), (F=143.5; P<0.001). There was no interaction between larval temperature and food (F=0.40; P=0.672).

### 3.2 Survival

**Figure 1.**
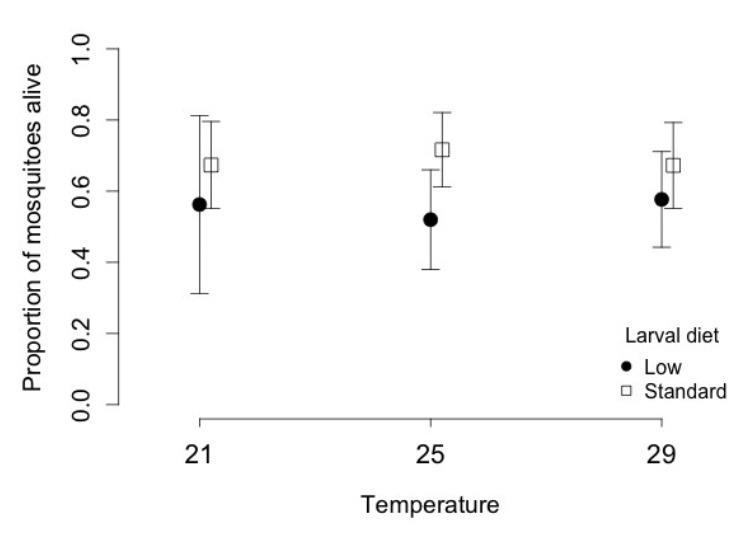
Proportion of mosquitoes that survived for 21 days after infection as a function of larval temperature and diet. Vertical lines represent the confidence intervals of the proportions.

Mosquitoes were more likely to survive for 21 days after infection (Figure 1), if they had been fed a standard diet as larvae (0.68; 95% confidence interval 0.61-0.75) rather than half the amount of food (0.55; 0.45-0.64), and larger mosquitoes had a greater chance of survival (Table 2). Survival time after infection increased by 1 day per 0.1mm of wing length: survival time after infection (days) = -18.4 + 10.5 * wing length. Neither temperature nor the interaction between food and temperature significantly affected survival (Table 2). At standard diet, the proportion of mosquitoes surviving was 0.67 (0.52-0.79) at 21ºC, 0.71 (0.590.81) at 25ºC and 0.67(0.53-0.78) at 29ºC. At low diet, it was 0.56 (0.30-0.79) at 21ºC, 0.52 (0.37-0.66) at 25ºC and 0.57(0.43-0.70) at 29ºC.

**Table 2.** Summary of statistical analyses.

		Survival		Infection		Vector competence	
Factor	df	c^2^	P	c^2^	P	c^2^	P
Temperature	2	0.14	0.92	1.18	0.55	0.87	0.64
Food	1	5.16	0.02	10.94	<0.001	13.83	<0.001
Temperature*Food	2	0.56	0.75	10.82	0.004	7.19	0.02
Wing length	1	19.17	<0.001	0.00	0.97	11.15	<0.001

### 3.3 Infection

**Figure 2.**
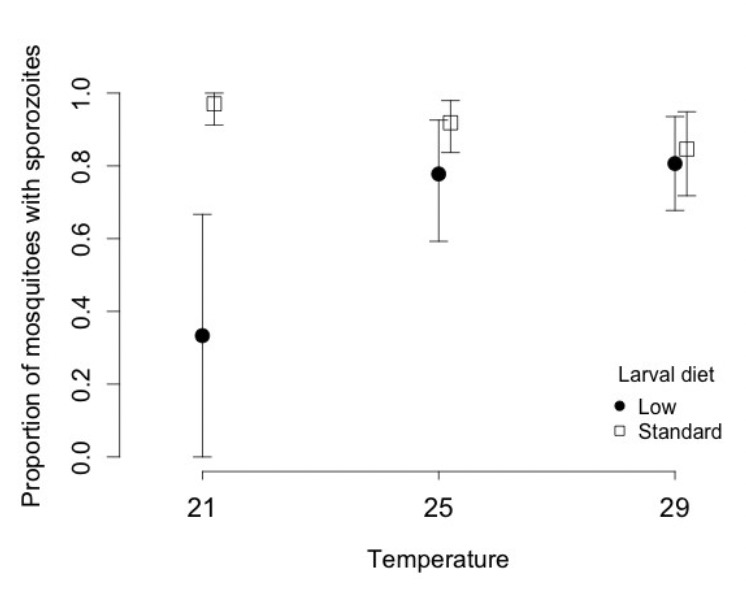
Proportion of mosquitoes with spor ozoites in their salivary glands 21 days post infection, as a function of larval temperature and diet. Vertical lines represent the confidence intervals of the proportions.

Surviving mosquitoes were more likely to harbour sporo-zoites (Figure 2), if they had obtained the standard diet (0.90; 0.84-0.95) rather than the low diet (0.73; 0.60-0.82). In contrast to its effect on survival, wing length had no significant effect on the probability of harbouring sporozoites in the salivary glands. Although there was no direct impact of temperature during larval development on spo-rozoite rate, temperature affected the impact of food (Table 2). The difference in sporozoite rate between the standard and the low diet decreased from 0.64 at 21ºC (standard 0.97, 0.82-0.99; low 0.33, 0.09-0.69), to 0.14 at 25ºC (standard 0.91, 0.79-0.97; low 0.77, 0.57-0.90) and 0.04 at 29ºC (standard 0.84, 0.68-0.93; low 0.80, 0.610.91).

### 3.4 Vector competence

**Figure 3.**
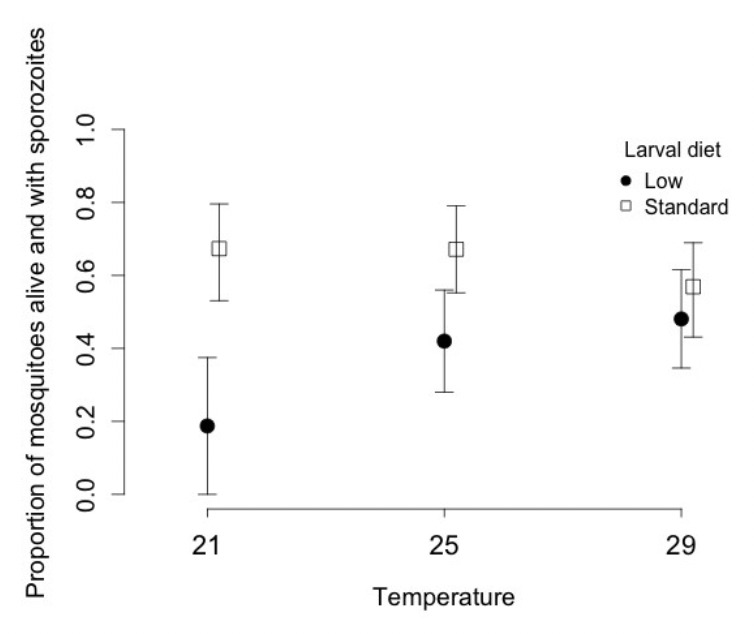
Proportion of mosquitoes that survived for 21 days post infection with sporozoites in their salivary glands, as a function of larval temperature and diet. Vertical lines represent the confidence intervals of the proportions.

Vector competence (the combination of survival up to dissection and the likelihood of harbouring sporozoites) was higher if larvae had been reared on the standard diet (0.63; 0.56-0.70) than on the low food diet (0.41; 0.32-0.50) and when the mosquitoes were larger (Table 2). The mean size of vector competent mosquitoes was 3.21 mm (± 0.02 SE) against 3.09 mm (± 0.02 SE) for non-vector competent ones. The effect of food was strongly affected by larval temperature (Table 2). After a standard diet, vector competence tended to decrease with increasing temperature from 0.67 (0.52-0.79) at 21ºC, to 0.56 (0.43-0.69) at 29ºC, but after a low diet vector competence increased from 0.18 (0.06-0.43) at 21ºC to 0.42 (0.28-0.56) at 25ºC and 0.48 (0.34-0.62) at 29ºC (Figure 3).

## 4 Discussion

Two aspects of larval environment – food and temperature – interacted to determine the vector competence of *A n. gambiae* for malaria. Low food decreased competence, but mostly at low temperature; at high temperature, food had a more limited effect. These results complement an earlier study [23] that showed that undernourishment of mosquitoes during larval development decreases the oocyst load (but not the prevalence) of malaria. They also show, however, that such results must be interpreted with caution, since the effect of one environmental factor can be influenced by other environmental factors.

In our experiment the effect on vector competence was due to a combination of survival during the parasite’s development and the proportion of the survivors that harboured sporozoites. The larval environment influenced both traits. Part of these effects was simply due to mosquito size. Undernourishment and high temperature both resulted in smaller adults (as is generally observed in invertebrates [30]) and smaller mosquitoes were generally less competent, corroborating earlier studies [24,31]. However, we found considerable effects of larval food and temperature after having controlled for the effects of size.

First, lower food availability decreased parasite and host survival. This may in part be due to the resources stored during the larval development [32] that can then be used, for example, to increase survival as adult. Although lower levels of food generally increase the lifespan of healthy individuals in a variety of mosquito species [33–35] and *An. gambiae* (unpublished data), it reduced the survival of infected mosquitoes due to parasite development. A possible explanation for this is that the resources acquired during larval development are critical to maintain an effective immune response and resistance against the parasite [36–38]. An alternative explanation is that, at low food diet, highly infected mosquitoes have a higher probability of dying than at standard diet. However, the resources do not appear to help control the parasite’s growth, as there is not less infection in well-fed mosquitoes. Rather, the lower parasite's survival in undernourished mosquitoes in our and other studies [23,39] suggests that these mosquitoes do not have acquired enough resources to support the parasite’s growth [32].

Second, the effect of temperature depended on the level of larval food and on the trait that was investigated. Adult temperature clearly affects the survival of mosquitoes and the developmental rate of parasites, and thus vectorial capacity, shown in several studies [1,12]. Larval temperature affects the size of the adults [20], which affects survival and perhaps parasite development. However, once we controlled for this indirect effect in our analysis, temperature had no effect on the survival of the mosquito. In contrast, low larval temperature impeded the parasite’s development in the adult, but only if larval food had been low. At 25°C and 29°C the influence of food was smaller, perhaps because the faster development of the mosquitoes at higher temperatures gave less time for the difference in food to affect the storage of resources. These results suggest that the mosquito’s ability to fight the parasite is weakest when the effects of temperature and food have conflicting effects on body size – low temperature increases adult size, but low food decreases it – and resources.

Since temperature affects metabolic and developmental rates, it is difficult to disentangle the effects of temperature and food availability. One possibility would be to use the same daily food regimes at the different temperatures. However, this would clearly lead to more total resources at low temperature. The best solution would be to match the resources to the physiological age of each individual, which is clearly not possible. We therefore decided to attempt to use a similar amount of total resources during larval development, based on our expectations obtained from earlier experiments for the developmental period. This of course does not preclude the possibility that our results are partly effects of variation of resources. Finally, we only considered the sporozoites rate at 21 days after infection, at which time almost all sporozoites will have been formed. It would also be interesting to see the influence of larval environment on the dynamics of the malaria parasite in the mosquitoes, as an increase or a decrease in the time before a mosquito become infectious is key to malaria transmission.

## 5 Conclusions

The larval environment influenced vector competence of adult malaria mosquitoes in a complex way. Thus, ideas and models about the role of environmental change on the transmission of malaria (and other vector-borne diseases) should include how the environment indirectly changes adult life-history by influencing larval development. In particular, we must consider the larval ecology to improve climate-based epidemiological modelling of malaria.
